# Plasma biomarkers of Alzheimer's disease in the continuum of dementia with Lewy bodies

**DOI:** 10.1002/alz.13653

**Published:** 2024-02-08

**Authors:** Patricia Diaz‐Galvan, Scott A. Przybelski, Alicia Algeciras‐Schimnich, Dan J. Figdore, Timothy G. Lesnick, Christopher G. Schwarz, Matthew L. Senjem, Jeffrey L. Gunter, Clifford R. Jack, Paul H Min, Manoj K. Jain, Toji Miyagawa, Leah K. Forsberg, Julie A. Fields, Rodolfo Savica, Jonathan Graff‐Radford, Vijay K. Ramanan, David T. Jones, Hugo Botha, Erik K. St Louis, David S. Knopman, Neill R. Graff‐Radford, Tanis J. Ferman, Ronald C. Petersen, Val J. Lowe, Bradley F. Boeve, Kejal Kantarci

**Affiliations:** ^1^ Department of Radiology Mayo Clinic Rochester Minnesota USA; ^2^ Department of Quantitative Health Sciences Mayo Clinic Rochester Minnesota USA; ^3^ Department of Laboratory Medicine and Pathology Mayo Clinic Rochester Minnesota USA; ^4^ Department of Radiology Mayo Clinic Jacksonville Florida USA; ^5^ Department of Neurology Mayo Clinic Rochester Minnesota USA; ^6^ Department of Psychiatry and Psychology Mayo Clinic Rochester Minnesota USA; ^7^ Mayo Center for Sleep Medicine Mayo Clinic Rochester Minnesota USA; ^8^ Departments of Neurology and Clinical and Translational Research Mayo Clinic Southwest Wisconsin La Crosse Wisconsin USA; ^9^ Department of Neurology Mayo Clinic Jacksonville Florida USA; ^10^ Department of Psychiatry & Psychology Mayo Clinic Jacksonville Florida USA

**Keywords:** Alzheimer's disease, Lewy body, mild cognitive impairment, PET biomarkers, plasma biomarkers, REM sleep behavior disorder

## Abstract

**INTRODUCTION:**

Patients with dementia with Lewy bodies (DLB) may have Alzheimers disease (AD) pathology that can be detected by plasma biomarkers. Our objective was to evaluate plasma biomarkers of AD and their association with positron emission tomography (PET) biomarkers of amyloid and tau deposition in the continuum of DLB, starting from prodromal stages of the disease.

**METHODS:**

The cohort included patients with isolated rapid eye movement (REM) sleep behavior disorder (iRBD), mild cognitive impairment with Lewy bodies (MCI‐LB), or DLB, with a concurrent blood draw and PET scans.

**RESULTS:**

Abnormal levels of plasma glial fibrillary acidic protein (GFAP) were found at the prodromal stage of MCI‐LB in association with increased amyloid PET. Abnormal levels of plasma phosphorylated tau (p‐tau)‐181 and neurofilament light (NfL) were found at the DLB stage. Plasma p‐tau‐181 showed the highest accuracy in detecting abnormal amyloid and tau PET in patients with DLB.

**DISCUSSION:**

The range of AD co‐pathology can be detected with plasma biomarkers in the DLB continuum, particularly with plasma p‐tau‐181 and GFAP.

## 1 BACKGROUND

Alzheimer's disease (AD) pathology at autopsy has been shown to co‐occur in more than half of the patients with dementia with Lewy bodies (DLB), together with the abnormal deposition of α‐synuclein.^1^ In vivo studies have confirmed that abnormalities in AD biomarkers occur in patients with DLB and its prodromal stages.^2^, ^3^ Therefore, biomarkers that detect the pathological abnormalities earlier in those patients with AD co‐pathology are needed.

The gold standard methods for obtaining in vivo biomarkers of AD are either the analysis of cerebrospinal fluid (CSF) or positron emission tomography (PET) imaging of amyloid beta (Aβ) and neurofibrillary tangle tau. However, these methods may be invasive or limited in accessibility. A possible turning point during the last few years has emerged with the development of AD biomarkers in plasma.^4^ Specific biomarkers of AD can be detected in plasma by analyzing the levels of Aβ 1‐40 (Aβ40), Aβ 1‐42 (Aβ42), and phosphorylated tau (p‐tau). Other measurable disease‐nonspecific biomarkers in plasma include neurofilament light (NfL) as a marker of neuronal injury and glial fibrillary acidic protein (GFAP) as a marker of astrocytic activation. A few studies in DLB show abnormal levels of plasma p‐tau, NfL, and GFAP in DLB, whereas the concentrations of plasma Aβ biomarkers are commonly within normal ranges.^5^, ^6^, ^7^, ^8^, ^9^ Previous studies have also shown that plasma biomarkers correlate with Aβ deposition on PET and that they can accurately discriminate between Aβ‐positive and Aβ‐negative DLB patients.^5^ Increasing evidence suggests that these abnormalities in plasma biomarkers may be present early in the continuum of the disease.^7^ However, data on plasma biomarkers in the prodromal stages of the disease are limited and correlations with Aβ and tau PET biomarkers of AD along the entire continuum of DLB need to be explored further.

The clinical continuum of DLB can include a preclinical and early emergence of isolated rapid eye movement (REM) sleep behavior disorder (iRBD), a prodromal stage of mild cognitive impairment with Lewy bodies (MCI‐LB), with the concurrent or subsequent development of the other core clinical features of DLB, followed by the dementia stage of DLB.^10^ In this study, our main objective was to investigate AD pathology along the DLB continuum using plasma biomarkers. First, we determined the values of plasma Aβ42/40 ratio, p‐tau‐181, NfL, and GFAP in the groups within the DLB continuum (iRBD, MCI‐LB, and DLB) and compared the levels of these plasma biomarkers at each stage of the DLB continuum with a group of clinically unimpaired controls. Second, we tested the associations of plasma biomarkers with PET biomarkers of Aβ and tau depositions. Third, we studied the ability of each plasma biomarker to detect abnormalities in AD PET biomarkers. We tested the discriminant ability of each plasma biomarker to identify DLB patients who are Aβ positive (A+), tau positive (T+), or both (A+T+) according to their Aβ and tau PET biomarkers.

## 2 METHODS

### 2.1 Participants

We included patients with a clinical diagnosis of iRBD (*n* = 15), probable MCI‐LB (*n* = 37), and probable DLB (*n* = 70) enrolled during the years 2007 to 2020 to the Mayo Clinic Alzheimer's Disease Research Center (ADRC), a longitudinal research study of individuals recruited from the clinical setting at Mayo Clinic. All patients underwent a comprehensive clinical evaluation including a medical history interview, informant interview, mental status examination, neurological examination, and neuropsychological examination. For this study, we selected those patients who had concurrent blood draw and Aβ PET with a subset completing tau PET (*n* = 80). Clinically unimpaired participants (CU; *n* = 100) without cognitive, motor, or sleep disorders were selected as a control group from the Mayo Clinic Study of Aging (MCSA), which is an epidemiologic study of aging in Olmsted County, MN (USA). We balanced CU with patients on age and sex through frequency matching.

**RESEARCH IN CONTEXT**

1. **Systematic review**: The authors reviewed the literature using traditional sources (e.g., PubMed). Data on the detection of Alzheimer's disease (AD) co‐pathology in patients with dementia with Lewy bodies (DLB) using plasma biomarkers are limited. Whether abnormalities in plasma biomarkers could be identified at the prodromal stages of DLB, and their association with positron emission tomography (PET) biomarkers of AD need to be addressed.
2. **Interpretation**: In the DLB continuum, the range of AD co‐pathology can be detected with plasma biomarkers in DLB. Glial fibrillary acidic protein (GFAP), a biomarker of neuroinflammation, is elevated starting from the prodromal stage of mild cognitive impairment with Lewy bodies in association with increased brain amyloid deposition. Plasma phosphorylated tau (p‐tau)‐181 can identify A+T+ profiles at the latest stage of DLB. Plasma biomarkers of AD can contribute to screening and early diagnosis in the DLB continuum, which has implications in designing clinical trials.
3. **Future directions**: Testing the combination of these plasma biomarkers in prospective longitudinal studies would strengthen the accurate prediction of AD co‐pathology in DLB.

The diagnosis of iRBD was based on the Classification of Sleep Disorders‐III criteria (ICSD‐III), indicating complex vocal and motor behaviors during REM sleep typifying dream enactment behavior with polysomnography‐confirmed REM sleep without atonia, and no neurologic (motor or cognitive) features.^11^ For the diagnosis of MCI‐LB, patients were required to have impairment in one or more cognitive domains, preserved or minimally affected performance of complex activities of daily living, and two or more core clinical feature of DLB or one core clinical feature of DLB and a positive DaTSCAN (dopamine active transporter scan).^3^ The diagnosis of clinically probable DLB requires two or more of the core clinical features.^12^ Details on the clinical assessment of patient groups within the DLB continuum and CU are elsewhere.^13^ Briefly, evaluations included information obtained through clinical interview with a neurologist, a neurologic examination, and a neuropsychological assessment. We used the Mini‐Mental State Examination (MMSE) to assess patients’ global cognitive status and Clinical Dementia Rating Sum of Boxes (CDR‐SOB) to assess dementia severity. Assessments for the clinical features of DLB were detailed in previous reports from the ADRC cohorts.^13^, ^14^ Briefly, parkinsonism was based on the neurological examination as having at least two of the four cardinal features (tremors, rigidity, bradykinesia, and postural instability). The Unified Parkinson's Disease Rating Scale Part III (UPDRS‐III) was used to quantify the severity of parkinsonism.^15^ Visual hallucinations had to be fully formed, not restricted to a single episode, and not related to another medical issue, dementia, or treatment. The 4‐item Mayo Fluctuation Scale was used to determine the presence of cognitive fluctuations.^16^ Finally, probable RBD was considered to be present when sleep symptoms consisted of excessive phasic or tonic electromyography (EMG) activity during recorded REM sleep, a history of injurious or disruptive sleep behavior or documentation of abnormal behavior during REM sleep in the laboratory, and an absence of electroencephalography (EEG) epileptiform activity during REM sleep as indicated in the International Classification of Sleep Disorders‐II (ICSD‐II), diagnostic criteria B.^17^

### 2.2 Blood collection and plasma assays

Ethylenediamine tetraacetic acid (EDTA) plasma samples were collected from participants after an overnight fast. Samples were centrifuged, and 500 μL of plasma was aliquoted into polypropylene tubes and stored at −80°C until testing. Plasma Aβ40, Aβ42, GFAP, and NfL were measured using the Simoa Neurology 4‐Plex E Advantage kit (N4PE, item #103670). Plasma phospho‐tau 181 (p‐tau‐181) was measured using the Simoa pTau‐181 Advantage V2 kit (item #103714). Both kits were used per manufacturer's instructions and ran on a Quanterix Simoa HD‐X analyzer (Quanterix, Lexington, MA, United States). Briefly, after thawing and mixing, plasma samples were centrifuged for 5 minutes × 4000 *g*. Samples were diluted 1:4 using the instrument's onboard dilution protocol and tested in singlet. A 7‐point calibration curve and sample concentrations were determined on the Simoa HD‐X Analyzer software using a weighting factor of 1/y^2^ and a 4‐parameter logistic curve fitting algorithm for p‐tau‐181. The N4PE test used 8‐point calibration curves with 1/y^2^ weighting; a 4‐parameter logistic fitting algorithm was used for NfL and GFAP, whereas a 5‐parameter logistic fitting algorithm was used for Aβ40 and Aβ42. Two levels of quality control material were run in duplicate with each batch following the assay calibrators. Inter‐assay imprecision for the quality control material (expressed as % coefficient of variation) were as follows: Aβ40, 5% and 3% at approximate concentrations of 16 and 117 pg/mL; Aβ42, 4% and 7% at approximate concentrations of 5.5 and 31 pg/mL; GFAP, 7% and 7% at approximate concentrations of 181 and 3702 pg/mL; NfL, 12% and 14% at approximate concentrations of 21 and 432 pg/mL; and p‐tau‐181, 6% and 5% at approximate concentrations of 3.7 and 119 pg/mL.

### 2.3 PET imaging

Aβ PET imaging was performed with Pittsburgh Compound B (PiB) and tau PET with ^18^F‐Flortaucipir (AV‐1451). For PiB scans, 560 MBq (range, 390‐681 MBq) ^11^C‐PiB was injected followed by four, 5‐minute dynamic frames after a 40‐minute ^11^C‐PiB uptake period. ^18^F‐Flortaucipir scans consisted of the injection of 387 MBq (range, 315‐407 MBq) ^18^F‐Flortaucipir followed by an 80‐minute uptake period. T1‐weighted magnetic resonance imaging (MRI) examinations were performed at 3T MRI and used in the PET data processing pipeline for anatomic segmentation and labeling. We used two, in‐house fully automated image‐processing pipelines to analyze PiB PET and ^18^F‐Flortaucipir PET images. Briefly, PET images were rigidly aligned to the individual's T1‐weighted MRIs using Statistical Parametric Mapping, version 12 (SPM12). MRIs were previously segmented with Unified Segmentation in SPM12 using population‐optimized priors from the Mayo Clinic Adult Lifespan Template (MCALT).^18^ After processing, the global standardized uptake value ratio (SUVr) of ^18^F‐Flortaucipir and PiB was calculated by normalizing the median uptake value in the cortex to the median uptake in the cerebellar crus gray matter. The global PiB retention SUVr was obtained for a standardized meta‐region including the prefrontal, orbitofrontal, parietal, temporal, anterior and posterior cingulate, and precuneus cortical regions. Global ^18^F‐Flortaucipir SUVr values were calculated for a composite of regions that reflects the tau PET AD signature. The tau AD signature comprises entorhinal, amygdala, parahippocampal, fusiform, inferior temporal, and middle temporal cortical regions. As described previously, SUVr values were classified as abnormal using a cut point of ≥1.48 for PiB and a cut point of ≥1.25 for ^18^F‐Flortaucipir.^19^

A voxel‐based analysis was conducted to test the relationship of each plasma biomarker with PiB and ^18^F‐Flortaucipir SUVr in the group of patients within the DLB continuum (iRBD+MCI‐LB+DLB; *n* = 122). We used a regression model including plasma Aβ42/40, p‐tau‐181, NfL, or GFAP as predictor and age as covariate in SPM12. Maps of significant associations were displayed at *p* < 0.05 level after correcting for multiple comparisons using false discovery rate error correction (FDR).

### 2.4 Statistical analyses

We report demographic and clinical characteristics using means and standard deviations (SDs) by clinical groups for continuous variables and counts and percentages for categorical variables. Overall group differences were analyzed with one‐way analysis of variance (ANOVA) for continuous variables, and chi‐square tests for categorical variables. Pairwise group comparisons to controls were done with Tukey tests. Because we were not interested in a universal null hypothesis, only few comparisons between each clinical group against the control group and did not want to inflate the probability of a Type II error, we did not adjust for multiple comparisons in these analyses. Rothman (1990) and Perneger (1998) provide good summaries for this reasoning.^20^, ^21^ PiB SUVr, ^18^F‐Flortaucipir SUVr, GFAP, NfL, and p‐tau‐181 were analyzed with natural log transformations due to skewness based on visual assessments. Age‐adjusted Pearson correlations were used for the associations of each AD plasma biomarker with PiB and ^18^F‐Flortaucipir SUVr. Age‐adjusted Pearson correlations were performed for the whole DLB continuum group of patients (iRBD+MCI‐LB+DLB), and for each group separately. Next, we performed multiple linear regression models within the DLB continuum group testing for interactions between PiB and ^18^F‐Flortaucipir SUVr while also adjusting for age with each plasma biomarker. Finally, receiver‐operating characteristic (ROC) curves comparing each plasma biomarker within the DLB group provided the area under the curve (AUC) for Aβ and tau PET positivity. AUC, sensitivity, and specificity were used to determine each plasma biomarker performance in identifying individuals who were classified as Aβ positive (A+), tau positive (T+), and both A+T+ according to the standardized PET biomarkers cutoffs. In all the analyses, statistical significance was deemed at *p* < 0.05 (two‐tailed).

### 2.5 Data availability

Data that support the findings of this study are available from the corresponding author upon reasonable request.

## 3 RESULTS

### 3.1 Demographic and clinical characteristics

Demographic, clinical, and cognitive characteristics of participants in the DLB continuum and CU groups are summarized in Table 1. Among the DLB continuum groups, the proportions of men were higher in the MCI‐LB (97%) and DLB (84%) groups than in the iRBD group (67%; *p* = 0.033). As expected, patients with DLB had worse cognitive performance with lower MMSE (*p* < 0.001) and higher CDR‐SOB (*p* < 0.001) scores than the CU group. Patients with MCI‐LB also showed worse clinical severity with higher CDR‐SOB than CU (*p* < 0.001). Global cortical PiB SUVr was higher in patients with DLB compared to CU (*p* < 0.001) but did not differ between patients with iRBD and CU (*p* = 0.950), nor between MCI‐LB and CU (*p* = 0.100). ^18^F‐Flortaucipir SUVr did not differ between patient groups and CU (*p* = 0.061) but showed a trend of higher levels in the DLB group (*p* = 0.063).

**TABLE 1:** Characteristics of the cohort by clinical group.

	CU *n* = 100	iRBD *n* = 15	MCI‐LB *n* = 37	DLB *n* = 70	*p*‐Value^*^
Age, years	68.76 (10.01)	67.29 (7.85)	68.69 (8.72)	69.50 (8.47)	0.848
Males, *n* (%)^[Link]^, ^[Link]^	86 (86%)	10 (67%)	36 (97%)	59 (84%)	0.033
*APOE* ε4, *n* (%)	27 (29%)	5 (36%)	11 (30%)	29 (42%)	0.324
Education, years	15.57 (2.34)	16.73 (3.13)	16.03 (2.69)	15.66 (3.05)	0.406
MMSE^†^	29.02 (0.90)	28.13 (1.13)	27.27 (2.16)	21.81 (5.72)	<0.001
CDR‐SOB^[Link]^, ^[Link]^	0.06 (0.32)	0.23 (0.62)	1.66 (0.96)	5.56 (3.27)	<0.001
Chronic kidney disease, *n* (%)	10 (10%)	1 (67%)	2 (5%)	2 (3%)	0.325
Abnormal DaTSCAN, *n* (%)^§^	NA	4 (40%)	21 (72%)	29 (91%)	0.004
Amyloid positive, *n* (%)^†^	30 (30%)	4 (27%)	10 (27%)	40 (57%)	<0.001
PiB AD signature SUVr^†^	1.53 (0.39)	1.46 (0.25)	1.53 (0.37)	1.78 (0.48)	<0.001
Tau positive, *n* (%)	27 (27%)	3 (21%)	8 (31%)	16 (40%)	0.419
Tau AD signature SUVr	1.20 (0.09)	1.19 (0.08)	1.20 (0.10)	1.27 (0.21)	0.061
Visual hallucinations, *n* (%)	NA	0 (0%)	5 (14%)	39 (56%)	<0.001
Cognitive fluctuations, *n* (%)	NA	0 (0%)	16 (43%)	52 (74%)	<0.001
Parkinsonism, *n* (%)	NA	3 (20%)	31 (84%)	62 (89%)	<0.001
RBD, *n* (%)	NA	15 (100%)	34 (92%)	66 (94%)	0.523
Aβ 42/40	0.06 (0.02)	0.06 (0.01)	0.06 (0.02)	0.06 (0.01)	0.387
GFAP^[Link]^, ^[Link]^	90.86 (47.88)	98.36 (42.99)	128.15 (76.33)	154.28 (86.12)	<0.001
NfL^†^	22.46 (14.40)	20.79 (5.33)	22.15 (11.10)	31.98 (21.40)	<0.001
p‐tau 181^†^	1.88 (0.95)	1.87 (0.86)	2.19 (1.22)	2.75 (1.42)	<0.001

Abbreviations: Aβ, amyloid beta; AD, Alzheimer's disease; *APOE* ε4, apolipoprotein E ε4 allele; CDR‐SOB, Clinical Dementia Rating‐Sum of Boxes; CU, cognitively unimpaired; DaTSCAN, dopamine active transporter scan; DLB, dementia with Lewy bodies; GFAP, glial fibrillary acidic protein; iRBD, isolated rapid eye movement (REM) sleep behavior disorder; MCI‐LB, mild cognitive impairment with Lewy bodies; MMSE, Mini‐Mental State Examination; NfL, neurofilament light; PiB, Pittsburgh compound B; p‐tau, phosphorylated tau; RBD, REM sleep behavior disorder; SUVr, standardized uptake value ratio.

^*^ *p*‐values for differences between groups come from either ANOVA for continuous variables or a chi‐square test for categorical variables.

^†^Significant differences between DLB and CU (*p* < 0.05).

^‡^Significant differences between MCI‐LB and CU (*p* < 0.05).

^§^DaT scan was available for a total of 10 iRBDs, 29 MCI‐LBs, and 32 DLBs.

### 3.2 Plasma biomarkers in the DLB continuum

**FIGURE 1:**
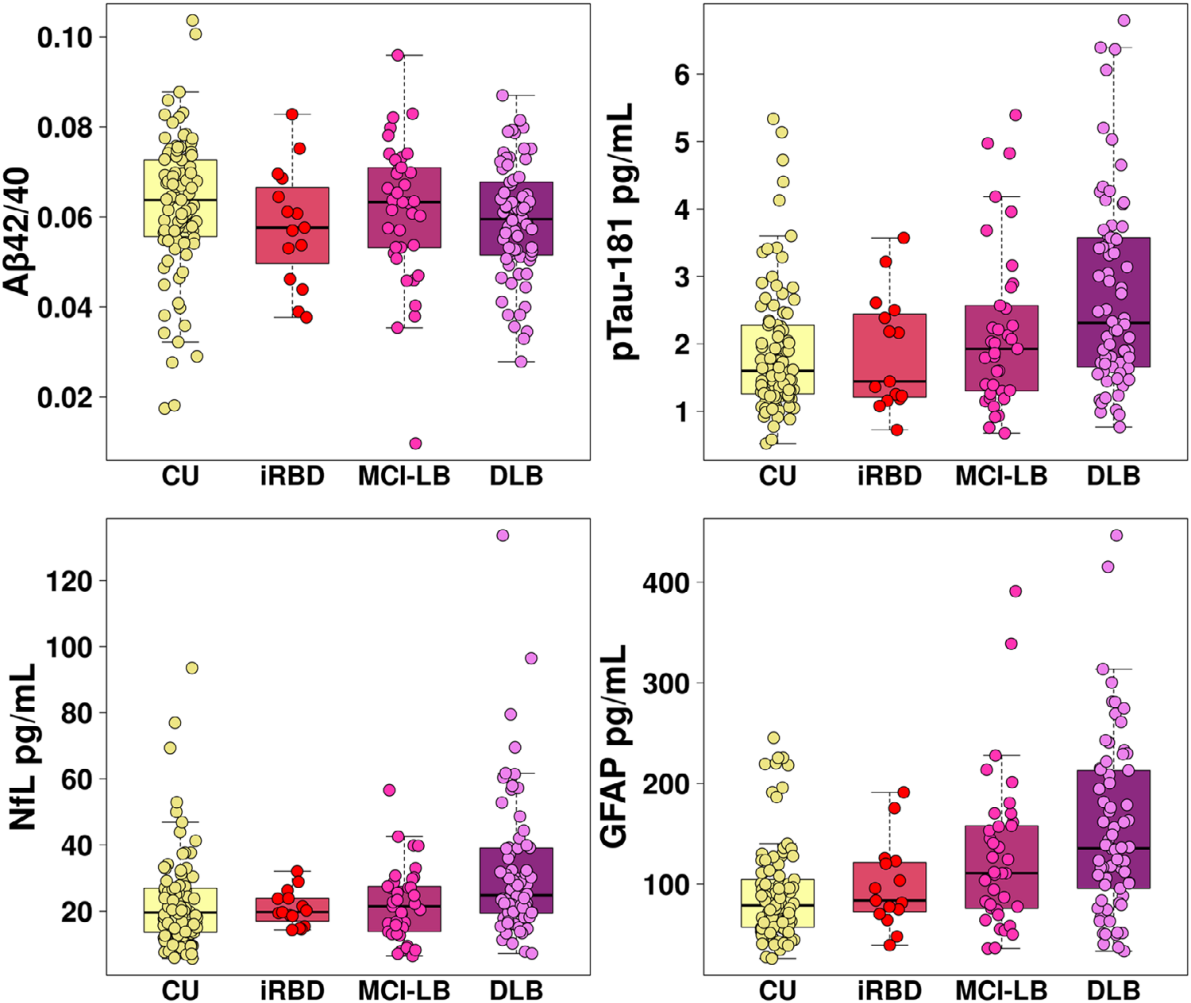
Concentrations of plasma biomarkers in the DLB continuum and controls. Aβ, amyloid beta; CU, cognitively unimpaired; DLB, dementia with Lewy bodies; GFAP, glial fibrillary acid protein; iRBD, isolated rapid eye movement (REM) sleep behavior disorder; NfL, neurofilament light; MCI‐LB, mild cognitive impairment with Lewy bodies; p‐tau, phosphorylated tau.

Figure 1 displays the plasma biomarkers of Aβ42/40, GFAP, NfL, and p‐tau‐181 among the CU, iRBD, MCI‐LB, and DLB groups. Compared to CU, patients with DLB had higher levels of GFAP, NfL, and p‐tau‐181 (*p* < 0.001). Patients with MCI‐LB showed higher levels of GFAP compared to CU (*p* = 0.014), whereas the levels of the other plasma biomarkers we studied were not different between MCI‐LB and CU. Patients with iRBD showed similar levels of all plasma biomarkers when compared to CU (Table 1). Groups did not differ in the levels of plasma Aβ42/40.

### 3.3 Correlation of plasma biomarkers with PET biomarkers

**FIGURE 2:**
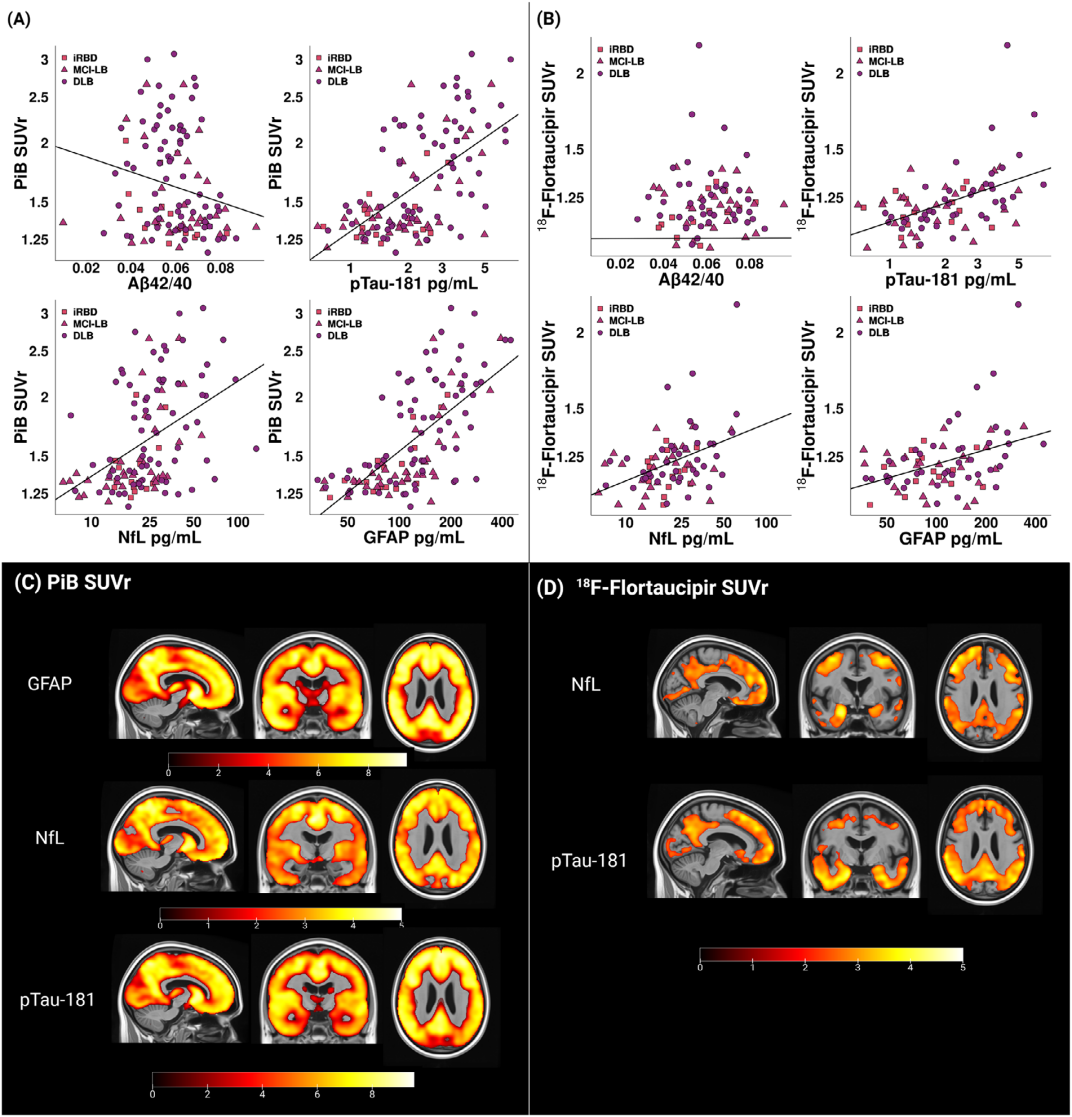
Associations of plasma biomarkers with amyloid and tau PET biomarkers. (A, B) Scatterplots of the correlations of plasma biomarkers with PiB SUVr and ^18^F‐Flortaucipir SUVr. (C, D) Voxel‐based analysis of the regional associations of plasma biomarkers with PiB and ^18^F‐Flortaucipir SUVr. Maps are displayed at the *p* < 0.05 level with the *t*‐values displayed in the color bar. Correction for multiple comparisons was applied with false discovery rate error correction. CU, cognitively unimpaired; DLB, dementia with Lewy bodies; GFAP, glial fibrillary acid protein; iRBD, isolated rapid eye movement (REM) sleep behavior disorder; NfL, neurofilament light; MCI‐LB, mild cognitive impairment with Lewy bodies; PiB, Pittsburgh compound B; p‐tau, phosphorylated tau; SUVR, standardized uptake value ratio.

Age‐adjusted Pearson's coefficients are displayed in Table 2 for the correlations of plasma biomarkers with PiB and ^18^F‐Flortaucipir SUVr. In the whole group of patients within the DLB continuum, higher PiB SUVr and higher ^18^F‐Flortaucipir SUVr were associated with higher levels of plasma p‐tau‐181, NfL, and GFAP (Figure 2A,B). We also studied the correlations of each plasma biomarker with PiB SUVr and ^18^F‐Flortaucipir SUVr in each clinical group within the DLB continuum (Table 2). In the iRBD group, higher PiB SUVr was associated with higher levels of plasma p‐tau‐181 and GFAP, whereas higher ^18^F‐Flortaucipir SUVr was associated with higher levels of plasma Aβ42/40. In the MCI‐LB group, higher PiB SUVr was associated with higher levels of plasma NfL, whereas none of the plasma biomarkers correlated with ^18^F‐Flortaucipir SUVr. In the DLB group, higher PiB and ^18^F‐Flortaucipir SUVr were associated with higher levels of plasma p‐tau‐181 and GFAP.

**TABLE 2:** Partial Pearson's correlations biomarkers with PET biomarkers.

(A) PiB SUVr								
			*Within the DLB continuum*					
	*DLB continuum*		iRBD		MCI‐LB		DLB	
	R	*p*	R	*p*	R	*p*	R	*p*
Aβ42/40	−0.18	0.052	−0.51	0.062	−0.09	0.599	−0.19	0.122
p‐tau‐181^*^	**0.56**	**<0.001**	**0.57**	**0.033**	**0.49**	**0.002**	**0.54**	**<0.001**
GFAP^*^	**0.63**	**<0.001**	**0.74**	**0.003**	**0.68**	**<0.001**	**0.58**	**<0.001**
NfL^*^	**0.33**	**<0.001**	0.32	0.269	**0.41**	**0.014**	0.22	0.066

(B) ^18^Flortaucipir SUVr								
			*Within the DLB continuum*					
	*DLB continuum*		iRBD		MCI‐LB		DLB	
	R	*p*	R	*p*	R	*p*	R	*p*
Aβ42/40	0.07	0.554	**0.65**	**0.017**	−0.050	0.812	0.010	0.951
p‐tau‐181^*^	**0.42**	**<0.001**	0.09	0.767	0.24	0.258	**0.52**	**<0.001**
GFAP^*^	**0.26**	**0.019**	0.10	0.752	0.05	0.807	**0.33**	**0.043**
NfL^*^	**0.29**	**0.010**	−0.05	0.861	0.35	0.086	0.30	0.066

Abbreviations: Aβ, amyloid beta; GFAP, glial fibrillary acidic protein; NfL, neurofilament light; p‐tau, phosphorylated‐tau.

* Plasma p‐tau‐181, GFAP, and NfL, as well as PiB and ^18^Flortaucipir SUVr were transformed for the analyses due to skewness.

Voxel‐based associations of PiB SUVr with plasma biomarkers of GFAP, NfL, and p‐tau‐181 in the whole group of patients within the DLB continuum are displayed in Figure 2C, demonstrating that higher PiB SUVr was associated with higher levels of plasma GFAP, NfL, and p‐tau‐181 across the whole cortex in a homogenous manner. Voxel‐based associations of ^18^F‐Flortaucipir SUVr with plasma biomarkers are displayed of Figure 2D, demonstrating that higher ^18^F‐Flortaucipir SUVr was associated with higher levels of plasma NfL and p‐tau‐181 in the frontal, middle temporal, and parietal cortices, with a relative sparing of the primary somatosensory cortex and occipital lobe.

**FIGURE 3:**
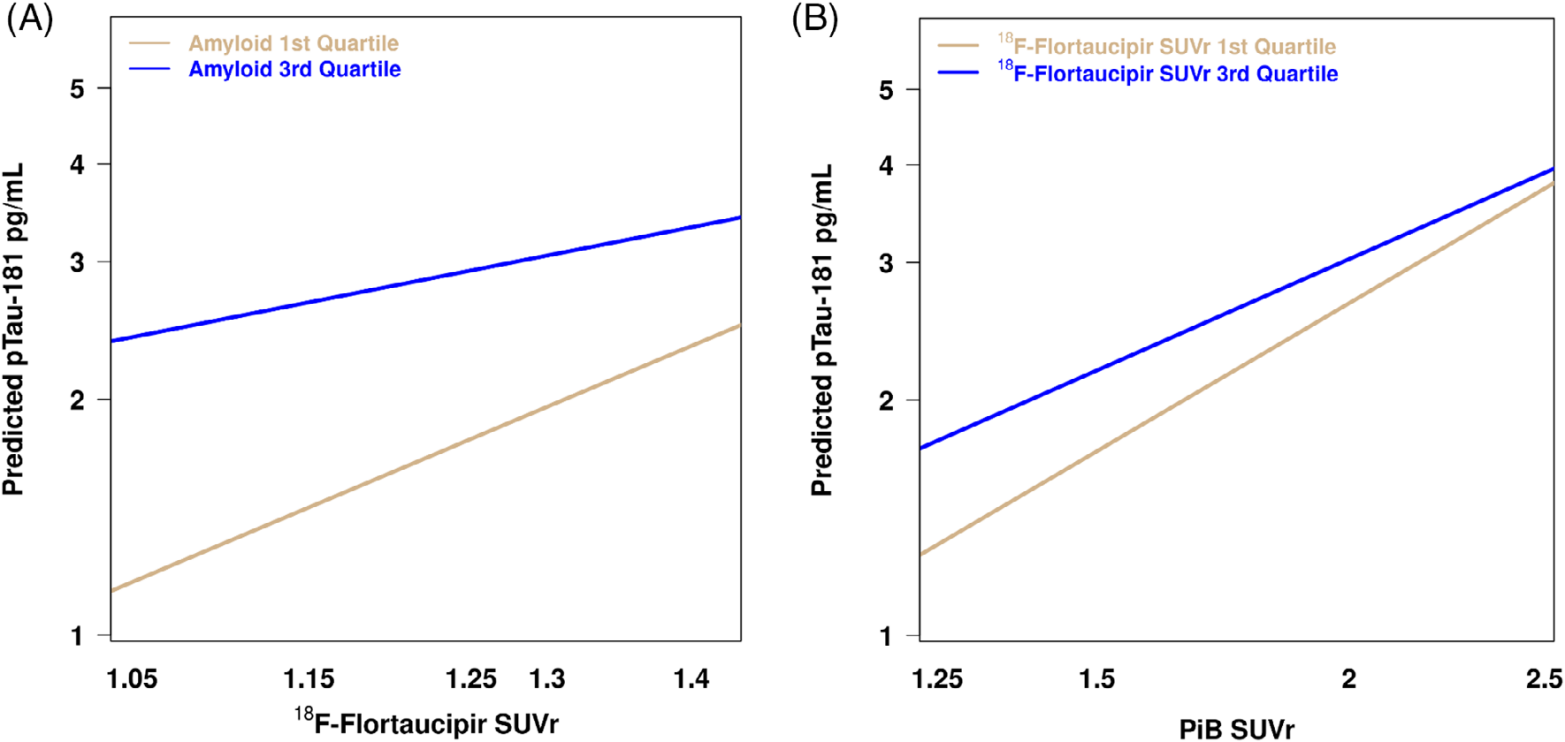
Interaction of PiB and ^18^Flortaucipir SUVr in the prediction of plasma p‐tau‐181. Data were stratified by (A) PiB SUVR and (B) ^18^Flortaucipir SUVR, which were stratified according to the first quartile, which indicated low Aβ or tau PET uptake, and to the third quartile, which indicates high Aβ or tau PET uptake. PiB, Pittsburgh compound B; pTau, phosphorylated tau; SUVR, standardized uptake value ratio.

Multiple regression analyses yielded an interaction between PiB and ^18^F‐Flortaucipir SUVr in their association with plasma p‐tau‐181 (*p* = 0.048). It showed that plasma p‐tau‐181 increases as the tau PET burden gets higher (Figure 3A), but this association is steeper in those cases with lower Aβ burden on PET (defined by PiB SUVr in the first quartile) than in those with higher Aβ burden (defined by PiB SUVr in the third quartile). Likewise, plasma p‐tau‐181 also increases as PiB SUVr reaches higher levels (Figure 3B), but those participants with lower tau PET burden (defined by ^18^Flortaucipir SUVr in the first quartile) had a greater increase in plasma p‐tau‐181 with higher PiB SUVr than those with high tau PET burden (defined by ^18^Flortaucipir SUVr in the third quartile). The interaction between PiB and ^18^F‐Flortaucipir SUVr was not significant for the rest of the plasma biomarkers (data not shown).

### 3.4 Discriminant ability of plasma biomarkers to identify Aβ and tau PET positivity

**FIGURE 4:**
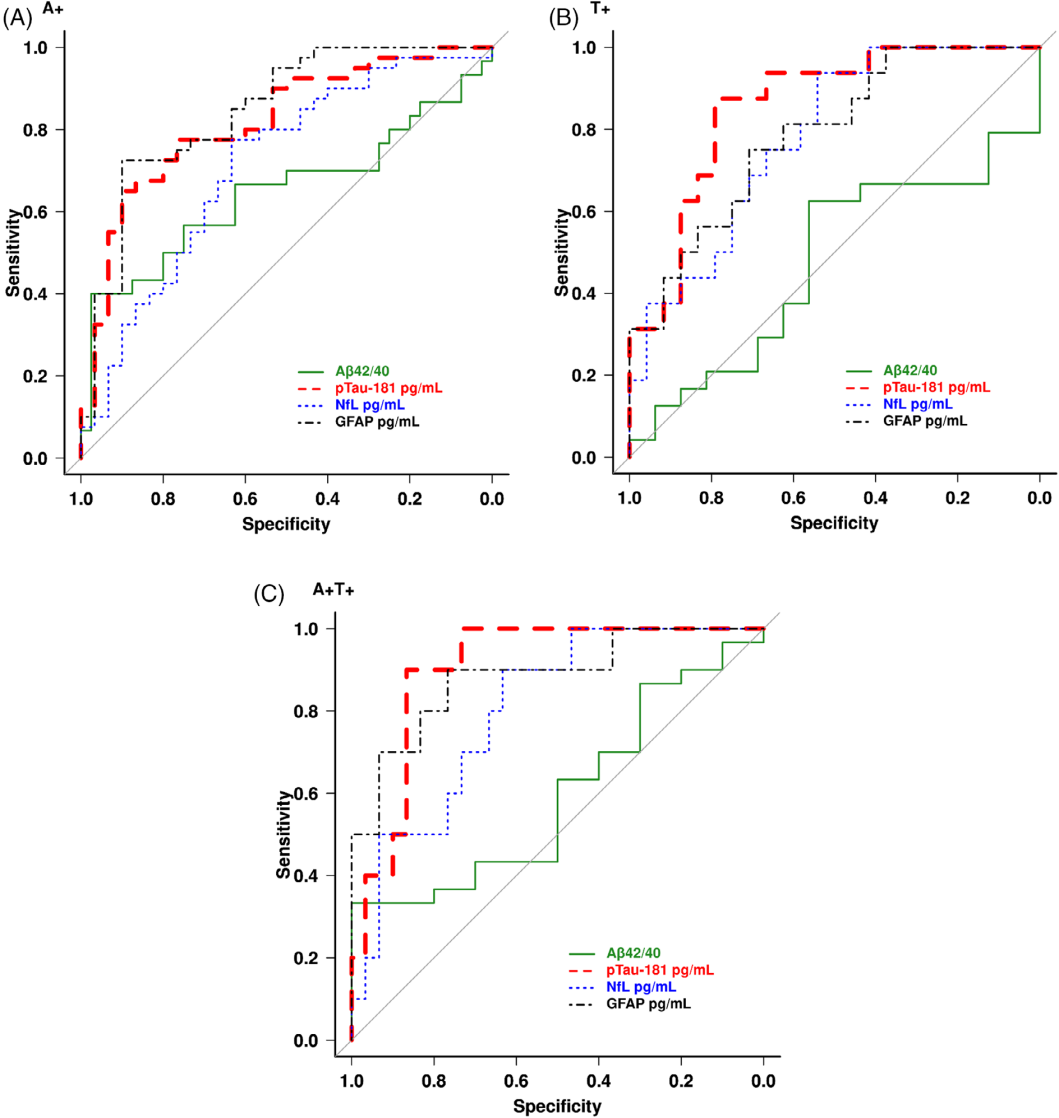
Performance of plasma biomarkers in identifying PET biomarkers positivity. Graphs show receiver‐operating characteristic (ROC) curves of each plasma biomarker for the discrimination of (A) A+ vs A– cases, (B) T+ vs T– cases, and (C) A+T+ vs A–T– cases. Aβ, amyloid beta; GFAP, glial fibrillary acid protein; NfL, neurofilament light; p‐tau, phosphorylated tau.

The potential for plasma biomarkers to differentiate abnormalities in PET biomarkers of Aβ and tau was investigated in the group of DLB patients only (*n* = 70). Based on a PiB SUVr cut point of 1.48, a total of 40 participants were Aβ positive (A+) and 30 were Aβ negative (A−). Forty participants also underwent tau PET scan with ^18^Flortaupicir. Based on a ^18^Flortaupicir SUVr cut point of 1.25, a total of 16 were tau positive (T+) and 24 were tau negative (T‐). There were also 16 patients who were classified as A+T+ according to both PiB and ^18^Flortaupicir SUVr cut points. ROC curves are displayed in Figure 4, showing the ability of each plasma biomarker to distinguish A+, T+, and A+T+ cases. Plasma GFAP had the highest accuracy (AUC = 0.85) in distinguishing A+ cases from A– cases with 73% sensitivity and 63% specificity (Figure 4A), whereas plasma p‐tau‐181 showed the highest accuracy (AUC = 0.86) in distinguishing T+ from T– cases with 79% sensitivity and 69% specificity (Figure 4B). In addition, plasma p‐tau‐181 showed the highest accuracy (AUC = 0.90) in distinguishing A+T+ cases from the rest of the cases with 87% sensitivity and 90% specificity (Figure 4C).

## 4 DISCUSSION

Our findings indicate that abnormal levels of GFAP in plasma can be detected as early as the prodromal stage of MCI‐LB within the DLB continuum, whereas abnormal plasma p‐tau‐181 and NfL are detected at the latest stage of DLB. Higher plasma GFAP was associated with higher Aβ burden from iRBD to DLB stages, and accurately identified brain Aβ pathology in DLB (A+). Higher plasma p‐tau‐181 was associated with higher Aβ and tau burden on PET at the DLB stage, either when both proteinopathies are present or alone. It also showed the highest accuracy in discriminating those DLB patients with an A+T+ profile. Therefore, plasma p‐tau‐181 could be used as a stand‐alone plasma biomarker of both pathologies in patients with DLB.

In this study, we quantified a panel of plasma biomarkers of disease‐specific biomarkers of AD (Aβ42/40, and p‐tau‐181) together with AD‐nonspecific biomarkers of neurodegeneration (NfL) and neuroinflammation (GFAP). For the disease‐specific biomarkers of AD, plasma Aβ42/40 did not differ from controls in any of the clinical groups within the DLB continuum, which is in line with previous studies.^6^, ^9^, ^22^ The lack of differences in plasma Aβ42/40 could be explained by the limitations of this plasma assay. Compared to CSF, plasma assays have substantial peripheral Aβ expression, which results in less‐pronounced decreases in the Aβ42 to Aβ40 ratio and a larger overlap of Aβ concentrations between A+ and A– individuals.^23^ Moreover, brain amyloidosis is present in 10% to 30% of individuals who are cognitively unimpaired, which decreases the probability of finding differences with clinical groups in Aβ plasma biomarkers.^24^, ^25^ Curiously, there was a counterintuitive correlation of higher Aβ42/40 with higher tau deposition in the iRBD group. This result might be due to a collider effect. Patients with iRBD are clinically normal, which reduces the probability of having AD pathology as reflected by in vivo biomarkers. This leads to false associations that reinforces this absence of pathology in the iRBD group. Overall, more investigation is needed to determine the best approach to measure plasma Aβ and its potential diagnostic value in DLB.

For plasma p‐tau181, we found abnormal levels at the DLB stage, which is consistent with previous studies.^6^, ^8^, ^9^, ^26^, ^27^ However, levels of plasma p‐tau‐181 were comparable to controls in both MCI‐LB and iRBD stages. Previous studies have shown increased levels of plasma p‐tau already at preclinical stages of AD^28^, ^29^ and DLB.^7^ Although our results are not statistically significant, a similar pattern was observed in our MCI‐LB group, where mean plasma p‐tau‐181 was higher in MCI‐LB individuals (mean = 2.19; SD = 1.22) than in controls (mean = 1.88; SD = 0.95).

Although abnormal plasma p‐tau‐181 was not identified until the DLB stage, plasma p‐tau‐181 correlated highly with PiB SUVr across the whole DLB continuum. This strong association between plasma p‐tau‐181 and Aβ deposition is in line with previous published studies in AD, showing that plasma p‐tau‐181 is a sensitive predictor of elevated brain Aβ on PET at preclinical stages, before observing widespread tau aggregates in the neocortex.^28^, ^30^, ^31^, ^32^, ^33^, ^34^ Therefore, our findings support the hypothesis that p‐tau‐181 can detect a neuronal reaction to initial Aβ aggregation in the very early stages of DLB, even when there are still no tau depositions. Plasma p‐tau‐181 also had a moderate correlation with ^18^Flortaucipir SUVr, but only at the latest stage of DLB.^35^, ^36^ We observed a significant correlation of higher plasma p‐tau‐181 with higher tau deposition in the frontal, temporal, and parietal regions, sparing the primary sensory and motor cortices, which reflects the typical topographical distribution of tau pathology in DLB.^37^, ^38^, ^39^ Of interest, at this late stage of DLB, plasma p‐tau‐181 was able to identify individuals with both brain Aβ and tau pathology (A+T+) with 90% accuracy, which is consistent with previous reports in AD.^40^

Of note, our multivariate analyses revealed that PiB and ^18^Flortaucipir SUVr interact with each other to predict increasing levels of plasma p‐tau‐181 in DLB. The association of higher plasma p‐tau‐181 with higher Aβ deposition is steeper at low levels of tau deposition than at high levels of tau deposition. Similarly, the association of higher plasma p‐tau‐181 with higher tau deposition is steeper at low levels than at high levels of Aβ deposition. This finding indicates that high levels of p‐tau‐181 can indicate Aβ or tau pathology alone or in combination. Because both proteinopathies contribute to plasma p‐tau‐181 variability, when Aβ or tau depositions are low, high levels of plasma p‐tau‐181 reflect underlying tau or Aβ pathology only, respectively. Similar results have been found in AD before.^40^ However, although the correlation of increased plasma p‐tau‐181 with Aβ deposition has been reported before in patients with DLB,^5^ the association with tau deposition, alone and in combination with Aβ pathology, was not explored yet in the DLB continuum. Therefore, our findings support current evidence suggesting that plasma p‐tau‐181 serves as an indicative marker for the underlying conditions leading to both defining proteinopathies of AD.^40^ Although plasma p‐tau‐181 lacks specificity to Aβ or tau depositions, it could become a cost‐ and time‐saving screening test for the evaluation of patients with suspected AD co‐pathology in DLB.

Within the biomarkers that were not specific to AD, findings on plasma GFAP levels stand out because of the early increase at the prodromal stage of MCI‐LB and its strong and consistent correlation with high PiB SUVr across the entire DLB continuum. Previous studies have already shown increased plasma GFAP in DLB and AD, and its correlation with Aβ deposition on PET.^5^, ^6^, ^22^, ^26^, ^41^ In the current study, we further demonstrated that higher plasma GFAP and its association with Aβ depositions is present during early stages of the DLB continuum. This is in line with a recent scheme of the changes in cognition and plasma biomarkers during AD development.^42^ It postulates that elevation in plasma GFAP occurs years before the elevation in p‐tau, NfL, and the onset of clinical symptoms.^42^ Our findings of abnormal levels of GFAP in MCI‐LB and its association with Aβ deposition starting from the iRBD stage supports the hypothesis that GFAP is, indeed, an early AD biomarker that increases before p‐tau or NfL in the course of the disease within the DLB continuum. Furthermore, plasma GFAP showed the highest accuracy in discriminating A+ from A– cases with an AUC up to 85%, which is similar to that reported in AD and DLB before.^5^, ^41^ Higher plasma GFAP levels also correlated with higher tau deposition, but only at the latest stage of DLB.^37^, ^38^, ^39^ Altogether, these associations suggest that plasma GFAP reflects an early response to Aβ aggregates, which triggers a neuroinflammatory state and promotes glial cell activation.^43^

In agreement with previous studies, we found increased levels of plasma NfL in DLB.^5^, ^8^, ^22^, ^44^ However, we did not find increased plasma NfL at iRBD or MCI‐LB when relative to controls. This is in contrast with recent studies showing that patients with probable MCI‐LB have increased plasma NfL as compared to controls.^8^, ^45^ The difference of results for plasma NfL in our study could be explained by the young age of our MCI‐LB group (mean age 68.9 years) when compared to the MCI‐LB groups included in previous studies (mean age range of 75.5–77.2 years).^8^, ^45^ A younger age of our MCI‐LB patients indicate that they may be at an even earlier stage within the DLB continuum, when there is still not enough neuronal injury to be detected with plasma NfL. This is consistent with evidence suggesting that plasma NfL increases progressively over the course of MCI in the AD continuum, reaching abnormal levels toward late prodromal stages of the disease.^22^, ^42^ Of interest, we found a strong correlation between higher plasma NfL and higher PiB SUVr only in the MCI‐LB group. Therefore, although NfL remains within normal ranges in our MCI‐LB group, the fact that NfL is associated with Aβ pathology might indicate that axonal damage has already occurred at this prodromal stage and that it is highly related with Aβ deposition.^46^ However, as the disease progresses to DLB, NfL might be a marker of both AD‐dependent and AD‐independent neuronal loss, being associated with other pathological processes such as Lewy body or cerebrovascular disease in DLB.^47^ These findings place plasma NfL as a good candidate to study the contribution of multiple pathologies to the disease in the DLB continuum, especially at the latest stages.

We acknowledge several limitations of our study. Diagnoses were determined using clinical criteria, and comparisons between plasma markers and neuropathological findings were not possible. Some of these clinical groups were relatively small, particularly the iRBD group, so detailed within‐group subanalyses were limited. We assessed four plasma biomarkers, but other candidates could yield different results, such as other described forms of p‐tau (e.g., p217, p231). Head‐to‐head comparisons have also shown that there are other individual assays for plasma Aβ40/Aβ42 with better diagnostic performance than the Quanterix analytes used in this study.^48^ However, the Quanterix platform used in this study is one of the only two platforms that are currently commercially available and where all plasma biomarkers can be run on the same platform.

Overall, our results highlight the importance of plasma biomarkers as good candidates to characterize the DLB continuum. Plasma GFAP can potentially be used to determine amyloid‐related pathology in prodromal stages of DLB, whereas p‐tau‐181 appears to be the most optimal plasma biomarker to detect both Aβ and tau pathologies at DLB. It makes p‐tau‐181 an ideal biomarker in relation to the biological definitions of AD co‐pathology in patients with DLB. This will be important for clinical trials targeting Aβ or tau or both in a subset of DLB patients with AD co‐pathology. It would also open the possibility for studying AD co‐pathology in DLB patients with limited CSF or PET data, such as large epidemiological studies or clinical cohorts from communities.

## CONFLICT OF INTEREST STATEMENT

The authors declare no conflicts of interest. Author disclosures are available in the supporting information

## CONSENT STATEMENT

The Mayo Clinic Institutional Review Board approved the study, and written informed consent on participation was obtained from all patients or an appropriate surrogate, as stipulated in the Declaration of Helsinki.

## Supporting information

Supporting information
